# The Phenotype and Functional Activity of Mesenchymal Stromal Cells in Pediatric Patients with Non-Malignant Hematological Diseases

**DOI:** 10.3390/cells9020431

**Published:** 2020-02-12

**Authors:** Zyrafete Kuҫi, Christiane Jordan, Sibylle Wehner, Jan Sörensen, Andrea Jarisch, Emilia Salzmann-Manrique, Lisa-Marie Pfeffermann, Thomas Klingebiel, Peter Bader, Selim Kuҫi

**Affiliations:** 1 University Hospital for Children and Adolescents, Division for Stem Cell Transplantation and Immunology, Goethe University Frankfurt am Main, 60528 Frankfurt am Main, Germany; zyrafete.kuci@kgu.de (Z.K.); sibylle.wehner@kgu.de (S.W.); jan.soerensen@kgu.de (J.S.); andrea.jarisch@kgu.de (A.J.); emilia.salzmann-manrique@kgu.de (E.S.-M.); thomas.klingebiel@kgu.de (T.K.); peter.bader@kgu.de (P.B.); 2 Institute for Transfusion Medicine and Immunohaematology, German Red Cross Blood Donor Service Baden-Württemberg-Hessen GmbH, Goethe University Hospital, 60528 Frankfurt am Main, Germany; c.jordan@blutspende.de (C.J.); l.pfeffermann@blutspende.de (L.-M.P.)

**Keywords:** mesenchymal stromal cells, non-malignant hematological diseases, thalassemia, sickle cell anemia, severe congenital neutropenia

## Abstract

As the biology of mesenchymal stromal cells (MSCs) in patients with non-malignant hematological diseases (NMHD) is poorly understood, in the current study we performed a basic characterization of the phenotype and functional activity of NMHD-MSCs. Bone marrow (BM) of patients with thalassemia major (TM) possessed a significantly higher number of nucleated cells (BM-MNCs)/mL BM than healthy donors (*P* < 0.0001), which however did not result in a higher number of colony forming units-fibroblast (CFU-F) per milliliter BM. In contrast, from 1 × 10^6^ BM-MNCs of patients with sickle cell disease (SCD) were generated significantly more CFU-Fs than from TM-BM-MNCs (*P* < 0.013) and control group (*P* < 0.02). In addition, NMHD-MSCs expressed significantly lower levels of CD146 molecule, demonstrated an equal proliferation potential and differentiated along three lineages (osteoblasts, chondrocytes and adipocytes) as healthy donors’ MSCs, with exception of TM-MSCs which differentiated weakly in adipocytes. In contrast to other NMHD-MSCs and healthy donors’ MSCs, TM-MSCs demonstrated an impaired in vitro immunosuppressive potential, either. Noteworthy, the majority of the immunosuppressive effect of NMHD-MSCs was mediated through prostaglandin-E2 (PGE2), because indomethacin (an inhibitor of PGE2 synthesis) was able to significantly reverse this effect. Our results indicate therefore that NMHD-MSCs, except TM-MSCs, may be used as an autologous cell-based therapy for post-transplant complications such as graft failure, graft-versus-host disease (GvHD) and osteonecrosis.

## 1. Introduction

Hematopoietic stem cell transplantation (HSCT) is the only effective treatment for a broad range of malignant and non-malignant hematological diseases such as severe form of beta thalassemia major (TM), sickle cell disease (SCD) and severe form of congenital neutropenia (SCN). Transplantation with hematopoietic stem cells of HLA-identical siblings is much more successful but as for only 25–40% of patients an HLA-identical sibling donor can be found [1] the other option remains transplantation of allogeneic hematopoietic stem cells. However, this kind of transplantation may be followed by many complications for example, graft failure, GvHD and osteonecrosis. Graft failure in non-malignant hematological diseases as SCD, thalassemia and SCN varies significantly from 2.3–52% according to different transplant settings [2,3,4,5]. Mesenchymal stem cells (MSCs) as an essential HSC niche component [6] and as good producers of hematopoietic growth factors [7,8] are an attractive therapeutic tool to enhance engraftment in malignancies [9,10] and non-malignant hematological disorders undergoing haplo-HSCT [11].

In addition, approximately 40% of transplanted patients with hematopoietic cells of HLA-identical siblings develop GvHD compared to 59% of patients transplanted with the hematopoietic cells of unrelated donors [12]. About 29–42% of patients with SCD, thalassemia and SCN depending on the patients’ age and source of hematopoietic stem cells develop GvHD after transplantation [2,3,4,5]. As MSCs express either constitutively or after their activation immunosuppressive molecules such as PGE_2_, IDO, HGF and TGF-β_1_ [13,14] they are used as a second-line therapy in the treatment of steroid-refractory acute GvHD in both children and adults [15].

Avascular necrosis or osteonecrosis (ON) is the most common and debilitating sequelae of conditioning regimens (following total body irradiation) for allogeneic HSC transplantation which appears in 4–19% of HSCT survivors [16]. Long, continuous exposure to corticosteroids during delayed intensification chemotherapy plays a pivotal role in the development of ON [17]. The disease itself may also induce development of osteonecrosis for example, 12.4% of patients with SCD have osteonecrosis [18], whereas 20% of patients with osteonecrosis suffered from SCD [19]. Autologous MSCs as cells with the potential to differentiate into osteoblasts and secrete angiogenic growth factors were used in the treatment of avascular osteonecrosis [20] and in the successful treatment of early-stage osteonecrosis of the femoral head in 100 patients [21].

Thus far, there are several reports on the MSCs isolated from the bone marrow of patients with aplastic anemia which do not differ from MSCs isolated from healthy donors, except their impaired differentiation into osteoblasts [22,23,24]. According to other reports, MSCs of patients with anemia are phenotypically and functionally equal to MSCs of healthy donors [25,26] or can even better differentiate into adipocytes [27,28]. In contrast to a handful of reports on MSCs from patients with anemia, there are only three reports on MSCs of patients with thalassemia. According to Yoon et al. [29], who reported data from one patient, there is no difference in the quality of MSCs as morphology, phenotype, proliferation, trilineage differentiation potential and immunosuppression in mixed lymphocyte reaction (MLR) compared to control. Aksoy et al. [30] reported that MSCs from thalassemia patients exerted also a higher proliferation potential than MSCs of healthy donors. Recently, Crippa et al. [31] confirmed a reduced frequency of primitive MSCs in β-thalassemia patients caused by increased reactive oxygen species (ROS) production in vitro which impaired MSC stemness properties.

Lebouvier et al. [32] reported that the bone marrow of SCD patients with osteonecrosis related to the disease possessed a higher number of mononuclear cells and CFU-Fs than patients who had osteonecrosis not-related to SCD, and healthy donors, as previously reported [33]. However, no differences in phenotype, potential to proliferate and differentiate as well as to suppress the immune reaction was observed compared to healthy control [32,33,34,35]. As the prevalence of SCN and CN is more than 10 cases pro million inhabitants [36] there are no data on MSCs generated from their bone marrow. There is only one report about MSCs generated from patients with neutropenia (chronic idiopathic neutropenia: CIN), which demonstrated no difference in morphology, phenotype, number of CFU-Fs, differentiation and immunosuppressive potential compared to control group [37].

As the biology of MSCs in NMHD is poorly understood, in the current study we asked whether from a small proportion of the bone marrow backup of patients with non-malignant hematological diseases is possible to generate functional autologous MSCs, which could be used for the treatment of complications of HSCT such as graft failure, GvHD and osteonecrosis.

## 2. Materials and Methods

### 2.1. Patients

In the current study, mesenchymal stromal cells were generated from the bone marrow aspirates of the posterior iliac crest of 21 children suffering from non-malignant hematological disorders with a median age of 11 years (range 3–15 years). Of 21 patients, ten of them suffered from thalassemia major (TM), nine from sickle-cell disease and two from congenital neutropenia. As a control were used MSCs generated from the bone marrow of 21 healthy donors with a median age of 12 years (range 3–16 years), using a protocol approved by the University of Frankfurt Institutional Review Board (UFIRB Nr. 19-472, 10 December 2019).

### 2.2. Isolation of Bone Marrow Mononuclear Cells (BM-MNCs)

Bone marrow aspirates were diluted 1:2 in phosphate buffered saline PBS and thereafter two volumes of samples were layered over 1 volume Ficoll (density: 1.077 g/mL) in a 50 mL conical tube and centrifuged at 700 × *g* for 25 min. Mononuclear cells were collected from the interface, then washed twice with PBS and centrifuged at 400 × *g* for 10 min.

### 2.3. Generation of Mesenchymal Stromal Cells (MSCs)

To generate MSCs, 4.3 × 10^6^ BM-MNCs were cultured in Dulbecco’s Modified Eagle’s Medium DMEM medium containing 10% MSC-qualified fetal bovine serum FBS (GIBCO/ Invitrogen, Darmstadt), in a T25 (25 cm^2^) tissue culture flask. Cells were incubated in an incubator with 5% CO2 and 95% humidity at 37 °C. After 48–72 h, the medium containing non-adherent cells was removed and replaced with fresh medium. The adherent cells were cultured for 10–14 days until the cells reached about 70–80% confluence. During this time the medium is changed every 3 days. MSCs were detached using trypsin and thereafter they were seeded at a density 2 × 10^3^ MSCs/cm^2^ and cultured for another 6–7 days until they reached the confluency. In the current study, the MSCs of passage 2 were used for differentiation and MLRs.

### 2.4. Estimation of the Frequency of Progenitor Cells for MSCs Using Colony Forming Units-Fibroblast (CFU-F) Assay

To determine frequency of progenitors for MSCs, different concentrations of BM-MNC (2 × 10^6^, 1 × 10^6^, 0.5 × 10^6^ and 0.25 × 10^6^) were plated onto T-25 with DMEM + 10% FBS. After 5 days the culture medium was removed and replaced with fresh medium. The medium was changed every 3rd day and dependently on generation of CFU-F they are enumerated on day 9–13. Colonies were stained with Giemsa’s solution. Cell clusters containing >50 cells were scored as CFU-Fs under an inverse microscope. The results are presented as number of colonies per 1 × 10^6^ BM-MNCs.

### 2.5. Immunophenotyping of Mesenchymal Stromal Cells (MSC)

MSC of passages 2 were labelled with fluorochrome-conjugated mouse anti-human antibodies against MSCs antigens CD90, CD73, CD105, CD146, CD271 (BioLegend, Koblenz, Germany) and hematopoietic antigens CD45, CD34 and CD14 as well as HLA Class II molecules (HLA-DR) (BioLegend, Koblenz, Germany). MSCs were incubated at 4 °C for 30 min, and after two wash steps with PBS + 0.2% bovine serum albumin BSA, flow cytometric analysis was performed on a FACSCalibur (Becton-Dickinson, Heidelberg) equipped with Macintosh software for data analysis (CellQuest). At least 50,000 events were acquired for each measurement.

### 2.6. Differentiation Potential of MSCs of Patients with Non-Malignant Hematological Diseases (NMHD)

#### 2.6.1. Differentiation into Osteoblasts

MSCs of passage 2 were plated in 6 well plates at a concentration 2 × 10^4^ MSCs/well in DMEM supplemented with 10% FCS for 6–7 days until they become a confluency of 90%. When the confluency was achieved the medium was removed and was replenished with medium for osteoblast differentiation Medium (StemMACS OsteoDiff Media, Media Miltenyi, Bergisch-Gladbach, Germany). On days 9–10 osteoblasts were identified by their cuboidal appearance and their association with newly synthesized bone matrix. Furthermore, committed osteogenic cells are characterized by expression of high levels of alkaline phosphatase (AP), an enzyme that is involved in the bone matrix mineralization, which can be detected using SIGMA FAST™ BCIP/NBT (5-bromo-4-chloro-3-indolyl-phosphate/nitro-blue tetrazolium) tablets as an insoluble substrate for the detection of alkaline phosphatase.

#### 2.6.2. Differentiation into Adipocytes

Mesenchymal stromal cells (MSCs) of passage 2 were cultivated in 6 well plates at a concentration 2 × 10^4^ MSCs/well in DMEM supplemented with 10% FCS for 6–7 days until they become a confluency of 90%. When the confluency was achieved, the medium was removed and was replenished with medium for adipocyte differentiation (StemMACS AdipoDiff, Media, Miltenyi, Bergisch Gladbach, Germany). The medium was changed every 3rd day. After 2–3 weeks, lipid vacuoles were developed and to visualize the adipocytes they are stained with Oil Red O solution (Millipore, Darmstadt, Germany) according to manufacturer’s instructions.

#### 2.6.3. Differentiation into Chondrocytes

To induce differentiation of MSCs into chondrocytes, 2.5 × 10^5^ cells of MSCs were resuspended in 1mL of pre-warmed NH ChondroDiff Medium from Miltenyi Biotec (Bergisch Gladbach, Germany). The medium was changed every third day and on day 24 the formed nodule was fixed in 3.7% neutral buffered formalin and then embedded in paraffin. Using a microtome, the nodule was cut into 5-μm thick tissue sections. After a deparaffinization step, tissue sections were stained with alcian blue (Merck, Darmstadt, Germany), a dye that stains glucosaminoglycans (an important component of the extracellular matrix produced by chondrocytes). The blue to bluish-green stained parts of tissue sections were evaluated by the microscope Olympus IX71 (Olympus, Hamburg, Germany). Differentiation capacity of MSCs into osteoblasts, adipocytes and chondrocytes was graded semiquantitatively by microscopy on the basis of the stained surface of differentiated MSCs by tissue-specific stainings.

### 2.7. Mixed Lymphocyte Reaction (MLR)

To study the immunosuppressive effect of mesenchymal stromal cells, isolated peripheral blood mononuclear cells (PB-MNCs) from buffy coats were stimulated with 0.4 µg/mL of anti-human CD3 and CD28 antibodies (clone HIT3a and CD28.2, BioLegend, Koblenz, Germany). In at least 6 wells, 1 × 10^5^ stimulated PB-MNCs/ well were plated in 96-well black plates with opaque flat bottom in 100 μL RPMI 1640 with 10% FBS. In certain wells 1 × 10^5^, 2 × 10^4^ of lethally irradiated (30 Gy) of MSC resuspended in 100 μL of RPMI 1640 with 10% FBS were added, which generated a MSCs to PB-MNCs ratio 1:1 and 1:5, respectively. In order to investigate the possible inhibitory effect of MSCs mediated by prostaglandin E2 (PGE2), the designated wells were treated with PGE2 inhibitor indomethacin (5 μM). The cells were incubated in an incubator at 37 °C in 5% CO_2_ and 95% humidity atmosphere. On day 6, the cells were labeled with BrdU (5-bromo-2′′-deoxyuridine) (Cell Proliferation enzyme-linked immunosorbent assay ELISA, BrdU chemiluminescent kit, Roche Applied Science, Mannheim) and further incubated for another 24 h in the incubator. The inhibitory effect of MSCs on proliferation of PB-MNCs on day 7 was presented as Relative Light Units (RLU/sec) using the luminometer 1420 Multilabel Counter Victor3, Perkin Elmer (Rodgau, Germany). In parallel, the same experimental design was set in another plate in order to determine cellular components, T-regs and concentration of PGE_2_ in the mixed lymphocyte reaction MLR supernatants. For estimation of PGE_2_ in MLR, supernatants of MLR were diluted 1:32 and then measured by using PGE2 FPIA kit (Enzo Life Science, Lörrach, Germany).

To examine changes in the phenotype of mononuclear cells in the presence or absence of third-party lethally irradiated MSC at an MSC:MNC ratio of 1:1, respectively 1:5 in MLR, we performed flow cytometric analysis on day 0 and day 7 as an end-point. In particular, to assess the percentage of T-regulatory cells, the MNC on days 0 and 6 were stained with monoclonal antibodies against CD3 (clone SK7), CD4 (clone RPA-T4) and CD25 (clone M-A251) antigens as well as CD45 (clone HI30). To identify Tregs within the MNC fraction, the gated CD3^+^ cells were examined for expression of CD4 and CD25 antigens. All antibodies were purchased from Biolegend (Koblenz, Germany).

### 2.8. Data Analysis

This is an exploratory study of phenotypic and functional characterization of MSCs from patients with non-malignant hematological diseases (TM, SCD and SCN) including a healthy donor control group. Descriptive statistics for categorical data were presented in absolute frequencies and percentages and for continuous data with median, maximum and minimum. Continuous variables were compared using the Wilcoxon-Mann-Whitney nonparametric test. Spearman’s or Kendall’s correlation was used to evaluate the possible correlation between the number of BM-MNCs per 1 mL of bone marrow and the number of colony-forming units- fibroblast CFU-Fs in each study group, as appropriate. All tests were two-sided with a significance level of 0.05. This study should be considered hypothesis-generating; therefore no adjustments for multiple testing were made. Statistical analyses were performed with R version 3.6.1 (foundation for statistical Computing, Vienna, Austria) and GraphPad Prism version 6 (GraphPad Software, San Diego, USA).

## 3. Results

Characteristics of the patients’ and healthy donors, whose bone marrow was used to generate MSCs are presented in Table 1.

### 3.1. Patients with Thalassemia Contain more BM-MNCs per Milliliter Bone Marrow than Patients with Sickle Cell Disease (SCD) and Control Group

**Figure 1:**
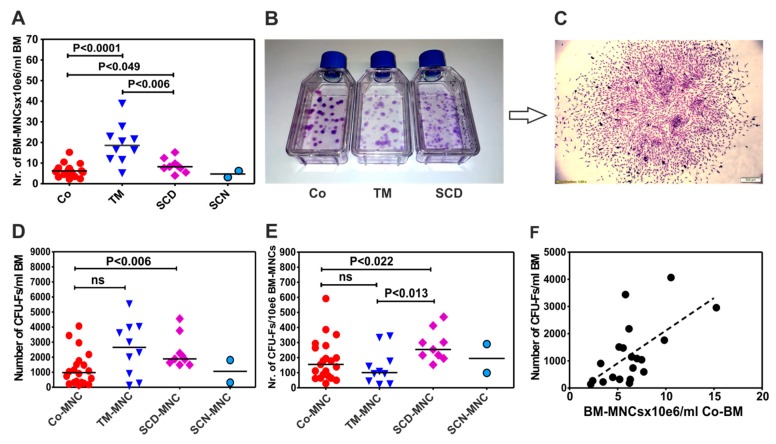
Cellularity and clonogenic potential of bone marrow mononuclear cells in patients with non-malignant hematological diseases (NMHD) (**A**) Cellularity of the bone marrow in patients with NMHD. (**B**) Morphology of colony-forming units-fibroblast CFU-Fs cultured in tissue culture flasks (**C**) A typical CFU-F, consisting of fibroblast-like cells. (**D**) Number of progenitor cells for mesenchymal stromal cells (MSCs) per milliliter BM aspirate of healthy donors and patients with NMHD. (**E**) Number of progenitor cells for MSCs per 1 × 10^6^ bone marrow mononuclear cells BM-MNCs of healthy donors and patients with NMHD. (**F**) Correlation between number of BM-MNCs/mL of the bone marrow of healthy donors and CFU-F. Differences between the groups were considered as statistically significant when *P* ≤ 0.05. Co: control; MNC: mononuclear cells; TM: thalassemia major; SCD: sickle cell disease; SCN: severe congenital neutropenia; BM: bone marrow.

Cell counting demonstrated that the bone marrow of patients with thalassemia possess a significantly higher number of mononuclear cells than healthy donors [18.57 × 10^6^ (5.125–38.83, N = 10) vs. 6.199 × 10^6^ (2.2–15.25), N = 20, *P* < 0.0001)]. They have also more mononuclear cells than SCD [8.24 × 10^6^/mL BM (3.94–15.23), N = 9; *P* < 0.006)] (Figure 1A). However, analysis of the number of progenitor cells for MSCs by using CFU-F assay (Colony-Forming Units-Fibroblast) (Figure 1B,C) demonstrated that bone marrow of thalassemia patients do not contain significantly more progenitor cells/mL bone marrow than bone marrow of control group (2649 CFU-F/mL BM (128.1–5534), N = 10) vs. (972.4 (143.05–4065.62), N = 20) (Figure 1D). In contrast, although the bone marrow of SCD patients did not contain significantly more BM-MNC/mL BM than control group, they generated significantly more CFU-F/mL BM than control group (1884 CFU-F/mL BM (1486–4554), N = 9; *P* < 0.006) (Figure 1D).

### 3.2. BM-MNCs of Patients with SCD Generate More CFU-Fs per 1×10^6^ BM-MNCs Compared to Patients with Thalassemia or Healthy Donors

Although patients with TM possessed a higher number of BM-MNCs, they did not generate significantly more CFU-Fs/1 × 10^6^ BM-MNCs than control group, (100.5 CFU-F/10^6^ BM-MNCs (22–343), N = 10 versus 155 CFU-F/10^6^ KM-MNCs (29–592), N = 21) (Figure 1E). In contrast, BM-MNCs of patients with SCD generated a significantly higher number of CFU-Fs/1 × 10^6^ BM-MNCs (254 (153–470), N = 9) than BM-MNCs of control group (155 (29–592), N = 21, (*P* < 0.02)) and patients with thalassemia (100.5 (22–343), N = 10, *P* < 0.013) (Figure 1E).

Further, we asked whether the higher number of mononuclear cells per milliliter of bone marrow correlates with the frequency of MSC progenitors (CFU-Fs) in each study group. Correlation analysis revealed a moderate but significant (confidence interval from 0.1224 to 0.8012, rho = 0.54, *P* < 0.01, N = 20) in the control group (Figure 1F). In contrast, there was no significant correlation between the number of BM-MNCs/mL of bone marrow and the number of CFU-Fs in patients with TM and SCD (data not shown).

### 3.3. BM-MNCs of Patients with NMHD Demonstrate a Normal Generation and Proliferation Potential of Mesenchymal Stromal Cells (MSCs)

**Figure 2:**
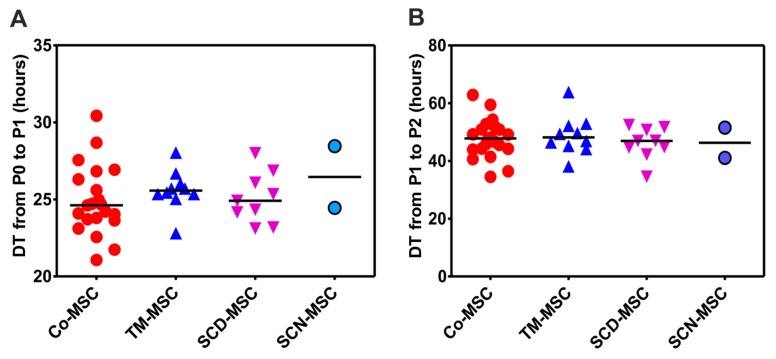
Proliferation potential of MSCs from different study groups at P0/P1 and P1/P2. (**A**) Time required for MSCs to reach one population doubling (doubling time = DT) at P0/P1 (**B**) Time required for MSCs to reach one population doubling (doubling time = DT) at P1/P2. Co: Control, TM: thalassemia major, SCD: sickle cell disease, SCN: severe congenital neutropenia.

Analysis of the BM-MNCs to generate MSCs at P0/P1 showed no difference between control group and patients with NMHD. One population doubling (PD) in the control group was reached after 24.62 h (21.07–30.43, N = 21) (DT = doubling time), whereas in patients it was reached after 25.34 h (22.8–28.46, N = 21) (data not shown). There was also no difference between control group and individual patients’ groups as well as no difference between patients’ groups themselves (Figure 2A). Similarly, there was no difference in the doubling time at P1/P2 between both control (47.81 h (34.53–62.88), N = 21) and patients’ groups together (47.45 h (34.56–63.84), N = 21) (data not shown) as well as between control group and individual patients’ groups (Figure 2B).

### 3.4. Phenotype of Mesenchymal Stromal Cells (MSCs) Generated by BM-MNCs of Patients with Non-Malignant Hematological Diseases (NMHD)

**Figure 3:**
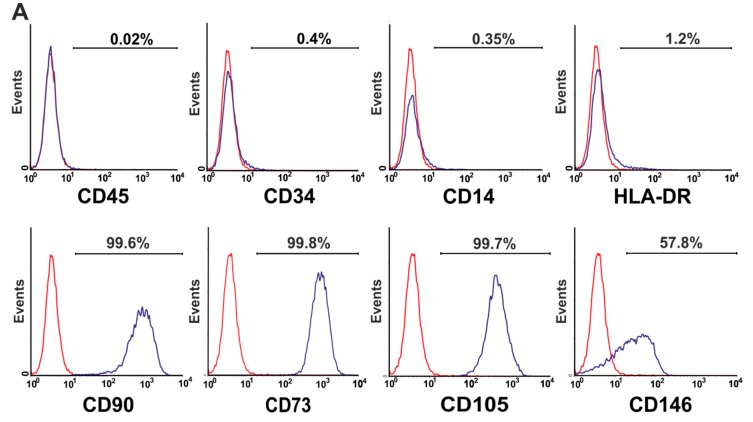

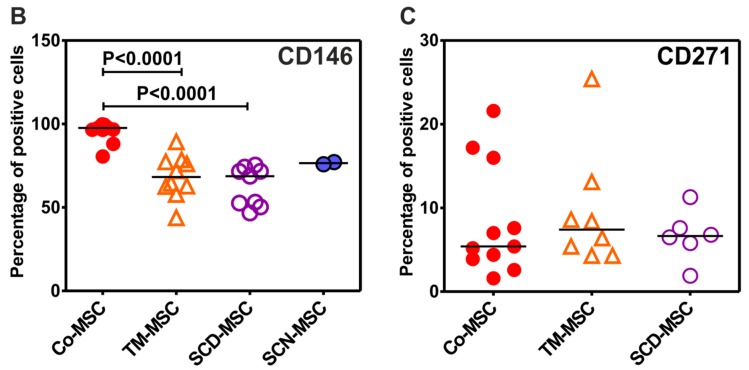
The phenotype and differences in the expression of CD146 and CD271 antigens on MSCs of patients with NMHD. (**A**) Phenotype of passage 2 MSCs in a patient with TM. MSCs were immunostained with fluorochrome-conjugated monoclonal antibodies and incubated at 4 °C for 30 min. After two wash steps with PBS + 0.2% BSA the stained cells were analyzed on a FACSCalibur (Becton-Dickinson, Heidelberg) equipped with Macintosh software for data analysis (CellQuest). At least 50.000 events were acquired for each measurement. (**B**) Expression of CD146 antigen on MSCs of passage 2 in patients with TM (68.2% (43.8–89.3), N = 10) and SCD (68.7% (46.7–75.4) N = 9)) was significantly lower than in MSCs of healthy controls (97.65% (80.6–99.8), N = 12, *P* < 0.0001). (**C**) Passage 2 MSCs of patients with non-malignant hematological diseases expressed similar levels of CD271 antigen as MSCs of healthy donors. Co: Control, TM: thalassemia major, SCD: sickle cell disease, SCN: severe congenital neutropenia.

Mesenchymal Stromal Cells (MSCs) of all patients with TM, SCD and CN express typical MSC-markers and lack the expression of hematopoietic markers in accordance with the minimal release criteria for MSCs as suggested by the ISCT-Committee [38] (Figure 3A). However, we found significantly lower levels of CD146 on MSCs of patients with TM and SCD, compared to MSCs of healthy donors (Figure 3B). In addition, no difference in the expression of CD271 antigen on MSCs of patients with TM and SCD and MSCs of healthy donors was observed (Figure 3C).

### 3.5. Trilineage Differentiation Potential of Mesenchymal Stromal Cells (MSCs) of Patients with Non-Malignant Hematological Diseases (NMHD)

Mesenchymal Stromal Cells (MSCs) of Non-Malignant Hematological Diseases (NMHD) differentiate equally well as healthy donors into osteoblasts, adipocytes and chondrocytes. However, thalassemic MSCs in addition to normal differentiation into osteoblasts and chondrocytes show a considerably weaker differentiation into adipocytes (Table 2).

### 3.6. Immunosuppressive Effect of Mesenchymal Stromal Cells (MSCs) of Patients with Non-Malignant Hematological Diseases (NMHD)

**Figure 4:**
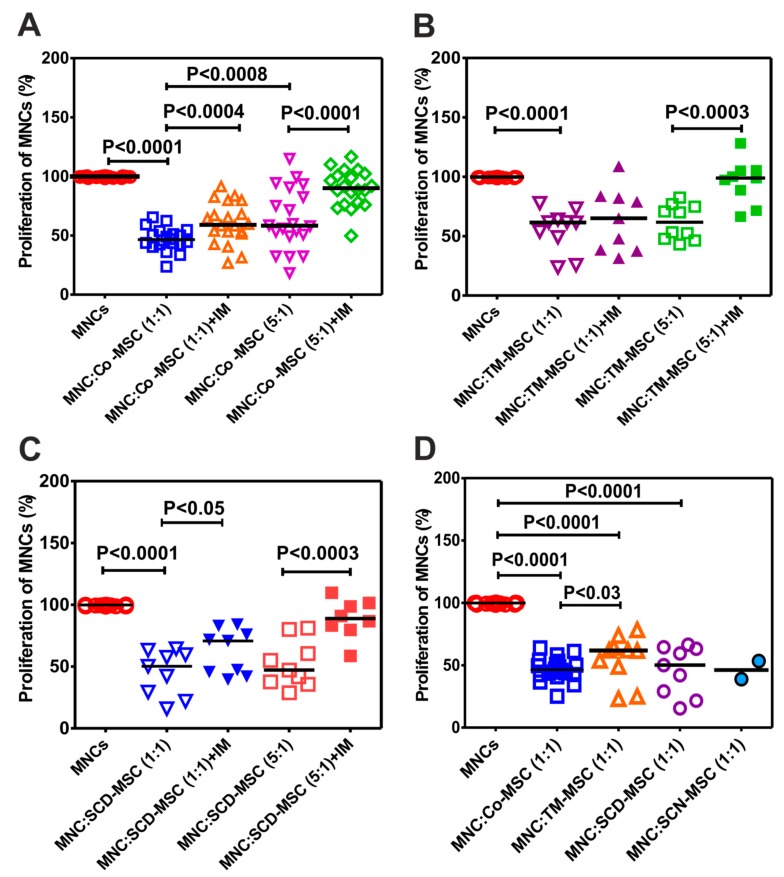
Immunosuppressive potential of MSCs from patients with NMHD and healthy donors (**A**) Healthy donor MSCs at different ratios with allogeneic MNCs (1:1 and 1:5) and the effect of indomethacin. (**B**) Inhibitory effect of MSCs from thalassemic patients. (**C**) inhibitory effect of MSCs of patients with SCD and (**D**) MSCs of patients with NMHD compared to control MSCs (only ratio 1:1) Differences between the groups were considered as statistically significant when *P* ≤ 0.05. Co: Control, TM: thalassemia major, SCD: sickle cell disease, SCN: severe congenital neutropenia.

In the current study we asked whether MSCs from patients with NMHD, in addition to their proliferative and differentiation potential, are also able to suppress proliferation of T cells in mixed lymphocyte reaction in vitro. As presented in Figure 4A, MSCs of healthy donors were able to inhibit proliferation of stimulated MNCs with anti-CD3/CD28 antibody. The inhibition has been shown to be dependent on the number of MSCs (more at the MNC:MSC ratio 1:1 compared to 5:1, *P* < 0.0008). In addition, indomethacin, an antagonist substance of prostaglandin E2, was able to significantly reverse the inhibitory effect of MSCs at both MNC:MSC ratio 1:1 and 5:1, *P* < 0.0004 and *P* < 0.0001, respectively), suggesting the role of PGE2 as a mediatory molecule of this effect. This prompted us to assess whether this effect is dose-dependent and if it can be reversed with the indomethacin in patients with NMHD. Indeed, when MSCs of thalassemic patients were used at the MNC:MSC ratio 1:1 they were more effective in suppressing proliferation of allogeneic MNCs than at the ratio 5:1, without reaching the significance. The reversal of this effect by indomethacin was more effective at the ratio 5:1 than 1:1, indicating the involvement of another mediator molecule than PGE2 at this ratio (Figure 4B). Similar potential demonstrated MSCs of patients with SCD, with the only difference that the inhibitory effect of their MSCs was significantly reversed in both MNC:MSC ratios 1:1 and 5:1. (Figure 4C). We asked whether MSCs of patients with NMHD behave in the same manner as MSCs of healthy donors. Indeed, our results show that MSCs of patients with SCD and SCN were as capable as healthy MSCs to inhibit proliferation of stimulated MNCs with anti-CD3/CD28 antibodies (Figure 4D). Interestingly, MSCs of patients with thalassemia showed a significantly impaired potential to inhibit this proliferation, compared to control (*P* < 0.03) (Figure 4D).

**Figure 5:**
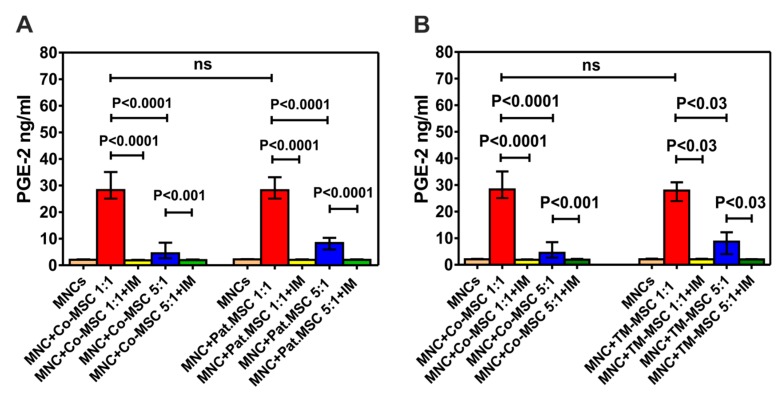
Concentration of PGE2 in the supernatants of MLR with MSCs from patients with NMHD and healthy donors. (**A**) Comparison of the PGE2 values in patients’ group with the control group (**B**) Comparison of the PGE2 values in patients with thalassemia and control group. Co: Control, Pat. MSC: Patients’ MSCs, IM: Indomethacin, TM: Thalassemia Major.

PGE2 molecule is one of the major mediator molecules which is involved in mediating the inhibitory effect of MSCs on the proliferation of allogeneic MNCs. Estimation of the concentration of this molecule in the supernatants of MLRs (co-culture of MSCs with MNCs) revealed that MSCs of patients secrete PGE2 at the same extent as the control group (28.29 ng/mL (23.93–47.7), N = 9). In addition, indomethacin as an inhibitor of PGE2 synthesis was able to significantly decrease the levels of this molecule at both MSC:MNC ratios (1:1 and 1:5) of control group and patients with NMHD (Figure 5A). Similar results from the analyzed supernatants were obtained when concentrations of PGE2 in MLR with thalassemia MSCs were compared with that of control group (Figure 5B). As expected, MNCs or MSCs alone secrete very low amounts of PGE2 (2.08 ng/mL (1.67–2.21) versus 1.62 ng/mL (1.61–1.64)], respectively).

**Figure 6:**
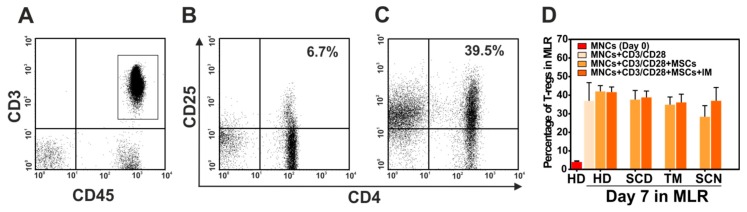
T-regulatory cells in mixed lymphocyte reaction MLR with MSCs of patients with non-malignant hematological diseases and healthy donors. (**A**) Dotplot of CD3+CD45+ T cells which were determined by flow cytometry after staining of mononuclear cells with anti-CD45/CD3/CD4/CD25 antibodies on day 0 in MLR. (**B**) Percentage of CD24+CD25+ cells after gating on CD45+CD3+ lymphocytes on day 0 and 7 (**C**). (**D**) The percentage of T-regs in each study group on day 7 of MLR. HD: healthy donors; SCD: sickle cell disease; TM: thalassemia major; SCN: severe congenital neutropenia.

In addition to PGE2, T-regulatory cells or T-regs are a subset of T cells which may mediate the inhibitory effect of MSCs on proliferation of T cells. Flow cytometric analysis of the cellular components in MLR on day 7, demonstrated an expansion of T-regs in stimulated MNCs with CD3/CD28 antibody either in presence or absence of MSCs in both patients with NMHD or healthy donors, compared to day 0 (Figure 6A–C). No difference in the levels of T-regs in the presence of patients’ MSCs or MSCs from healthy donors was observed. Interestingly, indomethacin was not able to reduce the level of T-regs, indicating that the expansion of these cells is not mediated by PGE2 molecule which is released from MSCs (Figure 6D).

## 4. Discussion

Mesenchymal stromal cells are one of the most attractive cell-based therapy options. In recent years, there has been increasing interest in the generation of functional MSCs from patients with different diseases in order to use them in autologous setting for example, in the treatment of rheumatoid arthritis [39], chronic osteoarthritis [40], Crohn’s disease [41], aGvHD and cGvHD [42]. According to these studies, there were no differences between MSCs generated from patients and healthy donors. However, MSCs of patients with non-malignant hematological diseases have been less well studied. In contrast to MSCs generated from patients with aplastic anemia (AA), which have been thoroughly investigated [22,23,24,25,26,27,28], MSCs from patients with thalassemia and SCD are poorly characterized [29,30,31,32,33,34,35], whereas MSCs generated from patients with congenital neutropenia are not studied at all. In the current study, we show for the first time that bone marrow of thalassemia patients contain a significantly higher number of mononuclear cells (BM-MNCs) than the bone marrow of healthy donors. However, they were not more potent in giving rise to a significantly greater number of CFU-Fs than BM-MNCs of other patients or control group. Nevertheless, BM-MNCs of thalassemic patients did not show impaired clonogenic efficiency compared to healthy donor counterparts, as recently reported [31]. This discrepancy may be explained by the fact, that this group used 1 × 10^5^/cm^2^ of selected CD34^−^ BM-MNCs to generate CFU-Fs, they performed colony counting on day 7 and their study group consisted of pediatric and adult patients. In contrast, we used 4 × 10^4^ /cm^2^ non-selected BM-MNCs and counted CFU-Fs on day 9–10. Moreover, MSCs from patients with SCD in our cohort of patients (N = 9), generated significantly more CFU-Fs than thalassemic and healthy donor MSCs as previously observed and reported [33]. According to Lebouvier et al. [32] patients with osteonecrosis related to SCD possess a higher number of BM-MNCs than patients with osteonecrosis not related to SCD and healthy control. In addition, these patients exerted a higher clonogenic efficiency especially SCD patients with osteonecrosis than patients with osteonecrosis not related to SCD and healthy controls. The presence of abnormal hemoglobin in their cells may require a high turnover of hematopoietic cells in the bone marrow and this disturbance may be the reason for the higher colony-forming efficiency (CFE) values in SCD patients.

Noteworthy, BM-MNCs of all patients with NMHD were able to give rise to MSCs. In addition, MSCs of patients with thalassemia and SCD demonstrated an equivalent proliferation potential as MSCs of healthy donors as reported by several groups [32,34,35]. Only one group reported that thalassemic MSCs proliferate more than MSCs of healthy donors [30], in contrast to Crippa et al. [31] who observed an opposite phenomenon. Flow cytometry analysis showed a significantly lower expression of CD146 antigen, as a more primitive cell marker, on MSCs of patients with TM, SCD and SCN compared to MSCs of healthy donors. Crippa et al. [31] reported same results for the patients with TM. To our best knowledge, we demonstrate for the first time significantly lower levels of CD146 antigen on MSCs of patients with SCD and SCN. The impaired expression of CD146 in the MSCs of patients with NMHD may be explained by the evidence that under normoxic conditions its expression is upregulated, whereas hypoxia induces a downregulation of its expression [43]. On this basis, one may speculate that the presence of abnormal hemoglobin in patients with NMHD may induce hypoxia, which in turn downregulates expression of CD146 antigen.

MSCs of patients with NMHD were able to equally differentiate into osteoblasts and chondrocytes as MSCs of healthy donors. Similar results for MSCs of patients with thalassemia [29,30] and SCD [32,34] were reported. Interestingly, we observed that MSCs of all four tested patients with thalassemia differentiated less well into adipocytes than MSCs of other NMHD and control group. In contrast, in MSCs of adult patients with anemia aplastica, an increased differentiation of adipocytes was observed [27,28], which was previously explained by the replacement of the marrow with fat cells [44]. According to Naveiras et al. [45], adult bone marrow also contains adipocytes, the numbers of which correlate inversely with the hematopoietic activity of the marrow. Very recently, Crippa et al. [31] by analyzing the induction of trilineage determination and tissue-specific genes found that thalassemia MSCs failed to efficiently differentiate into adipocytes and to form bone because of the significantly impaired expression of genes for lipid droplet formation in adipocytes (*LPL* and *FABP4)* and genes for bone formation (SPARC and COL1A2). In contrast to a reduced formation of adipocytes, MSCs of patients with thalassemia differentiated normally in bone (osteoblasts).

In the current study, we asked whether MSCs of patients with NMHD do have immunosuppressive properties. Indeed, our results show that MSCs of patients with SCD and congenital neutropenia were as effective in suppressing proliferation of T cells in MLR as MSCs of healthy controls. This effect was dose-dependent and could be partially reversed with indomethacin. Consent results with ours were reported in 2 single reports: one for SCD [35] and one for congenital idiopathic neutropenia [37]. In contrast, Yoon et al. [29] in their study with only one thalassemia patient found a normal immunosuppressive potential. Our results showed a significantly impaired immunosuppressive potential of TM-MSCs at the MNC:MSC ratio 1:1 compared to healthy control. This is in contrast with the recent findings of Crippa et al. [31] who revealed that TM-MSCs possess a normal immunosuppressive potential at high MSC:MNC ratio and a significantly impaired potential to inhibit MNC-proliferation at a low number of MSC (MSC:MNC = 1:200).

In order to identify the mechanism of the immunosuppressive potential, we asked whether PGE2, as a mediator molecule of MSCs immunomodulatory potential is also involved in the MSCs of patients with NMHD. Indeed, a major proportion of the immunosuppressive effect was mediated by this mediator, as indomethacin (a COX-1 and COX-2 inhibitor), was able to reverse this effect, as previously reported for bone marrow-derived MSCs [13,46]. To our knowledge, this is the first time to show that PGE2 molecule mediates the immunosuppressive effect of MSCs of patients with NMHD. Our findings are also in line with the study of Yañez et al. [47], who revealed that both adipose and bone marrow tissue-derived MSCs inhibited the maturation of myeloid-DCs and plasmocytoid-DCs. At the same time they found high levels of PGE2, which contributed to the inhibition of T lymphocyte proliferation and decreased production of inflammatory cytokines. Interestingly, indomethacin was able to reverse proliferation of T cells but was not able to restore production of inflammatory cytokines. Previous studies [48,49] demonstrated that higher PGE2 levels result in a higher immunosuppressive effect of MSCs, because PGE2 induces the development and expansion of immunomodulatory T-regs, either [13]. As in our study indomethacin was not able to reduce the level of T-regs, we assume that the expansion of T-regs was not mediated by PGE2 molecule but by stimulation of PB-MNCs with CD3/CD28 antibody as reported in previous studies [50,51]. However, because PGE2 appears to be one of the major mediators of the immunosuppressive effect of MSCs, it was proposed as a potency marker [48] to create an index of predicted efficacy for preparations of MSCs.

## 5. Conclusions

Our results indicate that NMHD-MSCs, except TM-MSCs, demonstrate functional properties comparable to healthy donors’ MSCs and therefore may be used as an autologous cell-based therapy for post-transplant complications such as graft failure, GvHD and osteonecrosis.

## Figures and Tables

**Table 1:** Characteristics of patients with non-malignant hematological diseases and healthy donors.

Gender	Healthy donors	TM	SCD	SCN-CN
N	21	10	9	2
Female, N (%)	12 (60)	5 (50)	5 (56)	2 (100)
Male, N (%)	9 (40)	5 (50)	4 (44)	0
Age in years: median (range)	12 (3–16)	10.5 (4–14)	14 (9–15)	7–11

N: number of donors; TM: thalassemia major; SCD: sickle cell disease; SCN-CN: severe congenital neutropenia-congenital neutropenia.

**Table 2:** Differentiation potential of MSCs from patients with NMHD and healthy donors. TM=thalassemia major, SCD = Sickle Cell Disease, SCN = Severe Congenital neutropenia, CN = Congenital Neutropenia, H.D. = Healthy donor, f = female, m = male, differentiation potential labeled with asterisks as: * weak, ** good and *** very good.

Patient’s ID.	Disease	Age (Years)/Gender	Osteoblasts	Adipocytes	Chondrocytes
			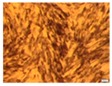	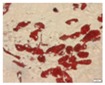	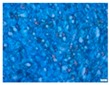
1	TM	9/f	***	*	***
2	TM	5/m	***	*	***
3	TM	13/m	**	*	***
4	TM	4/f	***	*	***
11	SCD	14/m	**	***	***
12	SCD	14/f	***	**	***
13	SCD	15/f	***	***	**
20	SCN	11/f	***	***	***
21	CN	7/f	***	**	***
**Healthy Donors’ ID**					
22	HD	8/m	**	***	***
23	HD	6/m	***	***	***
24	HD	13/m	***	***	***
25	HD	4/f	*	***	***
32	HD	12/f	***	***	***
33	HD	14/f	***	*	
34	HD	16/m	***	**	***
35	HD	13/m	***	**	***
41	HD	12/f	**	***	***
42	HD	8/f	***	***	***
